# Electrochemical Determination of Creatinine Based on Multienzyme Cascade-Modified Nafion/Gold Nanoparticles/Screen-Printed Carbon Composite Biosensors

**DOI:** 10.3390/s25134132

**Published:** 2025-07-02

**Authors:** Jialin Yang, Ruizhi Yu, Wanxin Zhang, Yijia Wang, Zejun Deng

**Affiliations:** 1 School of Materials Science and Engineering, State Key Laboratory of Powder Metallurgy, Central South University, Changsha 410083, China; 2 Hunan Key Laboratory for Super Microstructure and Ultrafast Process, School of Physics, Central South University, Changsha 410083, China

**Keywords:** creatinine, electrochemical biosensor, multi-enzyme cascade modification, gold nanoparticles

## Abstract

Creatinine serves as a crucial diagnostic biomarker for assessing kidney disease. This work developed portable non-enzymatic and multienzyme-modified electrochemical biosensors for the detection of creatinine based on commercial screen-printed carbon electrodes (SPCEs). The non-enzymatic creatinine sensor was constructed by the electrochemical deposition of AuNPs onto the surface of a pre-activated SPCE by electrochemical activation, followed by the surface modification of a Nafion membrane. The developed AuNPs/SCPE exhibited excellent reproducibility, and the proposed Nafion/AuNPs/SPCE sensor showed excellent detection sensitivity and selectivity toward creatinine. In comparison, the enzymatic creatinine biosensor was gradually established by the electrodeposition of a Prussian blue (PB) membrane on the optimal AuNPs/SCPE surface, followed by multi-enzyme cascade modification (which consisted of creatinine amidohydrolase (CA), creatine oxidase (CI) and sarcosine oxidase (SOx)) and drop-casting the Nafion membrane to stabilize the interface. The introduction of a PB interlayer acted as the redox layer to monitor the generation of hydrogen peroxide (H_2_O_2_) produced by the enzymatic reaction, while the Nafion membrane enhanced the detection selectivity toward creatine, and the multi-enzyme cascade modification further increased the detection specificity. Both non-enzymatic and enzymatic creatinine sensors could detect the lowest concentrations of less than or equal to 10 μM. In addition, the efficiency and reproducibility of the proposed composite biosensor were also confirmed by repetitive electrochemical measurements in human serum, which showed a positive linear calibration relation of peak currents versus the logarithm of the concentration between 10 μM and 1000 μM, namely, i_p_ (μA) = −7.06 lgC (μM) −5.30, R^2^ = 0.996. This work offers a simple and feasible approach to the development of enzymatic and non-enzymatic creatinine biosensors.

## 1. Introduction

Creatinine is primarily excreted by the kidneys and serves as a crucial biomarker for assessing renal function [1]. Clinically, elevated creatinine levels often indicate acute/chronic kidney injury or renal failure, highlighting the need for rapid, accurate, and portable detection methods [2]. Currently, the Jaffe colorimetric assay method and high-performance liquid chromatography (HPLC) are widely used in clinical laboratories for creatinine detection [3]. However, the Jaffe method is prone to false-positive results due to interference from proteins, glucose, and other substances in blood samples, while the HPLC technique needs expensive equipment that greatly limits its widespread use [4,5,6]. In addition, these conventional techniques typically require complex sample pretreatment and skilled personnel, making them unsuitable for point-of-care tests (POCTs) [7]. Consequently, the development of a cost-effective, user-friendly, and field-deployable creatinine detection method has become a key research focus [8,9].

Electrochemical biosensors, owing to their high sensitivity, rapid response, low cost, and miniaturization potential, have demonstrated significant promise in POCT applications [10,11,12]. Screen-printed carbon electrodes (SPCEs) and carbon paste electrodes are widely used as substrate electrodes for the detection of creatinine biomarkers by the subsequent modification of nanomaterials or bioreceptors, such as Cu NPs, Cu_2_O NPs, Au NPs, reduced graphene oxide (rGO), and enzymes for creatinine detection [13,14,15,16,17,18,19]. For instance, Chuang and coworkers developed a creatinine biosensor by the surface modification of Cu_2_O-Au nanohybrids onto the SPCE, achieving the accurate detection of creatinine levels in human serum. Bhat and coworkers developed an enzymatic amperometric sensor for creatinine by the enzymatic hydrolysis of creatinine to produce 1-methylhydantoin that was used as an indirect measure of creatinine levels [19]. Generally, non-enzymatic detection methods are susceptible to interference from coexisting substances such as uric acid and ascorbic acid in biological samples [20,21]. Existing creatinine sensors still face challenges, including enzyme deactivation, electrode surface fouling, and insufficient long-term stability [22].

Whether for non-enzymatic or enzymatic detection, most portable sensors are based on commercially available screen-printed carbon electrodes [23,24]. These commercial screen-printed carbon electrodes are often used directly without further processing, which typically results in low response currents (due to the gradual oxidation of the carbon surface during long-term storage). When such passivated electrodes are modified without pretreatment, both the sensor sensitivity and detection limit are substantially compromised. Consequently, the chemical or electrochemical activation of commercial SPCEs before functionalization has become essential for optimal performance.

This work comparatively presents two distinct sensor architectures, including a non-enzymatic sensor utilizing gold nanoparticles (AuNPs)-modified SPCEs and a multi-enzyme cascade creatinine sensor integrating creatinine amidohydrolase (CA), creatine oxidase (CI), and sarcosine oxidase (SOx). Before the construction of sensors, the SPCE substrate was electrochemically activated to counteract the fouling effects of storage-induced oxidation when stocked in vials. Regarding the enzymatic creatinine biosensor, Prussian blue (PB) membrane was electrodeposited onto the optimal AuNPs-modified SPCEs to act as the redox layer to monitor the generation of hydrogen peroxide (H_2_O_2_) produced by the enzymatic reaction, followed by the drop-casting modification of multienzymes. Both the non-enzymatic sensor and the enzymatic biosensors were finally drop-coated with a Nafion membrane to mitigate electrode fouling and stabilize the sensory interface.

## 2. Materials and Methods

### 2.1. Materials

Analytical grade reagents of sodium chloride, hydrochloric acid, sodium hydroxide, potassium ferricyanide, and potassium ferrocyanide were purchased from Sigma-Aldrich Co., Ltd., Shanghai, China. Creatinine was purchased from Heowns Bio-Technology Co., Ltd., Tianjing, China. Creatine amidinohydrolase, creatine amidinohydrolase, and sarcosine oxidase were purchased from Toyobo Co., Ltd., Shanghai, China. Nafion membrane was purchased from D&B Bio-Technology Co., Ltd., Guangzhou, China. Screen-printed carbon electrodes (product mode: C220) were purchased from Changsha SunJeen Electronic Technology Co., Ltd., Changsha, China. Human serum samples were obtained from healthy donors at the Second Xiangya Hospital of Central South University. Deionized water was used throughout the experiments.

### 2.2. Surface Modification of SPCEs

Commercial SPCEs were pretreated by electrochemical activation in acid (1 M H_2_SO_4_) or alkaline solution in the range of −1.6 V to 2 V. Then, Au NPs modification was achieved by the electrochemical reduction of 1 mM HAuCl_4_ at a constant potential of −0.5 V, during which different deposition times (50 s, 100 s, 200 s, 300 s, and 500 s included) were used to compare its effect on the electrochemical performance of the modified electrode, denoted as AuNPs/SPCE. For a non-enzymatic creatinine sensor, a Nafion membrane with different volumes was drop-coated onto the AuNPs/SPCE in order to increase its selectivity toward creatinine in the presence of a primary interfering agent (i.e., ascorbic acid).

### 2.3. Multienzyme Cascade Modification

Before the construction of the creatinine biosensor, Prussian blue was further electro-deposited onto the Au NPs/SPCE surface via cycling in the range of −0.15 V to 0.3 V in a solution of 2.5 mM K_3_Fe(CN)_6_, 2.5 mM FeCl_3_, 100 mM KCl, and 100 mM HCl. The as-prepared electrode was named PB/AuNPs/SPCE. To improve the selectivity of the PB/AuNPs/SPCE, the creatinine biosensor was constructed based on the principle of multi-enzyme cascade reaction, in which creatinine amidohydrolase (CA), creatine oxidase (CI), and sarcosine oxidase (SOx) were co-immobilized on the surface of the PB/AuNPs/SPCE via physical adsorption. The solution of creatinine hydrolase (8 U/μL dissolved in deionized water), creatine oxidase (4 U/μL dissolved in deionized water) and sarcosine oxidase (2 U/μL dissolved in deionized water) was mixed with glutaraldehyde (1% dissolved in deionized water) at a volume ratio of 1:1:1:3. The modified electrode was placed in a constant temperature and humidity chamber at 60% RH and 25 °C for 2 h to form a uniform film. Finally, a 0.5% Nafion solution was drop-coated onto the electrode to stabilize the modification layer. The as-prepared composite biosensor was stored in a 4 °C refrigerator for future use.

## 3. Results

### 3.1. Effects of Electrochemical Activation on the Electrochemical Performance of SPCE

**Figure 1:**
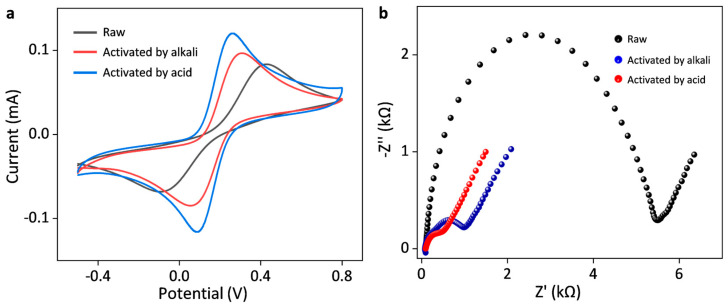
Electrochemical activation treatment of SPCEs. (**a**) Cyclic voltammograms and (**b**) electrochemical impedance spectroscopy of the raw SPCE and the activated SPCEs by concentrated alkali (1 M NaOH) and acid (1 M H_2_SO_4_) in a solution containing 5 mM K_3_[Fe(CN)_6_] and 5 mM K_4_[Fe(CN)_6_] with 0.1 M KCl as the electrolyte. The scan rate is 50 mV s^−1^, and the EIS tests were performed at open-circuit potential.

Commercial SPCEs usually encounter surface passivation caused by oxidation in the ambient environment. It is necessary to electrochemically activate those SPCEs before further measurements. Figure 1a shows cyclic voltammograms of the raw SPCEs and the activated SPCEs by concentrated alkali (1 M H_2_SO_4_) and acid (1 M NaOH) in a solution containing 5 mM K_3_[Fe(CN)_6_] and 5 mM K_4_[Fe(CN)_6_] with 0.1 M KCl as the electrolyte. Those activated SPCEs were performed by cycling in 1 M H_2_SO_4_ or 1 M NaOH for ten cycles at a potential range between −1.6 V and 2 V. After electrochemical activation, the peak currents of both activated SPCEs were significantly enhanced. The peak separation potential (Δ*E*_p_) of the activated SPCE by alkali and acid decreased to 0.175 V and 0.247 V from 0.515 V (obtained with raw SPCE). The SPCE activated by concentrated acid exhibited faster electrochemical kinetics and higher response currents compared to that by concentrated alkali. Electrochemical impedance spectroscopy (EIS) measurements evidenced similar enhancement in the basic electrochemical kinetics for those activated SPCEs compared to the raw one. The *R*_ct_ of the SPCE activated by acid and alkali were 0.41 kΩ and 0.89 kΩ, respectively, much smaller than that of raw SPCE (5.39 kΩ), indicating enhanced electrochemical reaction kinetics. Thus, subsequent electrochemical measurements were performed with the activated SPCE by concentrated acid.

### 3.2. Surface Modification of SPCEs by AuNPs and Their Resulting Electrochemical Performance

**Figure 2:**
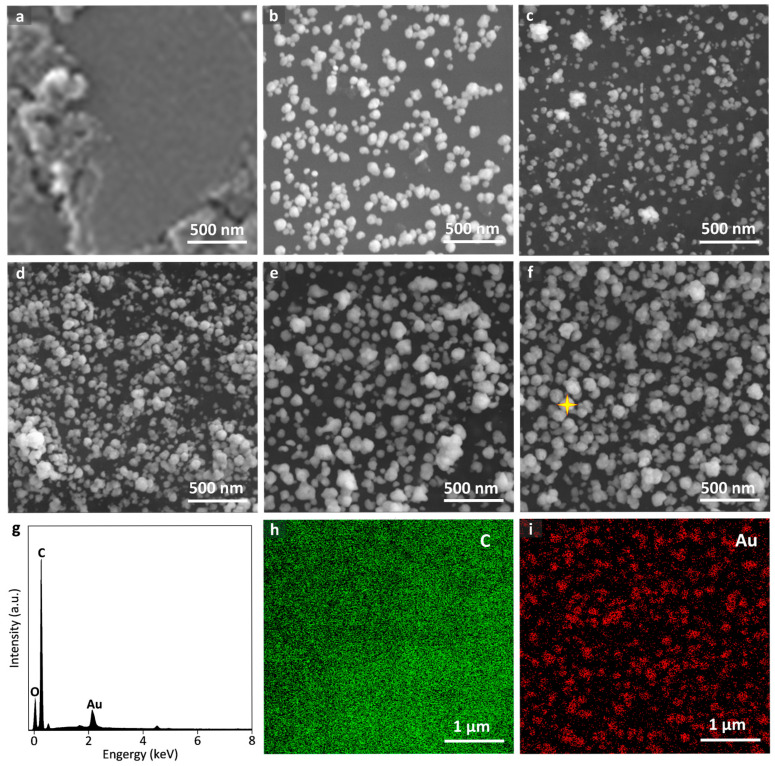
SEM images of (**a**) the raw SPECE and the modified SPCEs by the electro-deposition of AuNPs at different times of (**b**) 50 s, (**c**) 100 s, (**d**) 200 s, (**e**) 300 s, and (**f**) 500s. (**g**) Energy dispersive spectrum of the AuNPs modified SPCE sampled at the marked location of (**f**) (in yellow star). (**h**–**i**) Elemental distribution mapping analysis of the AuNPs modified SPCE with elements of carbon and gold.

SEM images of the raw SPCE and the AuNPs-modified SPCEs at different times of 50 s, 100 s, 200 s, 300 s, and 500 s are shown in Figure 2a–f, respectively. The unmodified SPCE exhibited an irregular flake-like stacking structure. Concerning the AuNPs-modified SPCEs, AuNPs preferentially electrochemically deposit on the uneven regions of electrode surfaces owing to the higher surface energy, which is beneficial for facilitating the nucleation of AuNPs. The size of the AuNPs ranged from 50 to 150 nm in diameter. As the deposition time increased, the size and the density of the AuNPs increased, forming the nanoclusters. The energy dispersive spectrum (EDS) confirmed the successful deposition of the AuNPs (Figure 2g), and an even element distribution of carbon and gold on the SPCE surface was observed in Figure 2h,i.

The basic electrochemical performance of AuNPs-modified SPCEs was systematically investigated, including heterogeneous electron transfer kinetics (*k*^0^), interfacial electron transfer resistance (*R*_ct_), and electrochemically active surface area (ECSA) [25,26]. According to the Nicholson method in which Δ*E*_p_ is a function of a dimensionless kinetic parameter (*ψ*), one can quantitatively obtain the *k*^0^ values based on the slope of the linear calibration relation of *ψ* as a function of the inverse square root of the scan rates (*v*^−1/2^), as depicted in Equation (1) [27,28]. For a quasi-reversible system (Δ*E*_p_ ≤ 200 mV), *ψ* is described by Equation (2), while for an irreversible system (Δ*E*_p_ > 200 mV) it is described by Equation (3).

(1) $$\psi =\frac{{\left(D_{O}/D_{R}\right)}^{\alpha /2}k^{0}}{\left(\pi D_{O}nF/RT\right)}v^{-1/2}$$

when Δ*E*_p_ ≤ 200 mV,

(2) $$\psi =\frac{\left(-0.6288+0.0021\times \Delta E_{p}\times n\right)}{\left(1-0.017\times \Delta E_{p}\times n\right)}$$

when Δ*E*_p_ ˃ 200 mV,

(3) $$\psi =2.18\times {\left(\beta /\pi \right)}^{1/2}\exp\left[-\left(\beta^{2}\times \frac{F}{RT}\right)\Delta E_{p}\right]$$

where *ψ* is the dimensionless kinetic parameter, *D*_O_ and *D*_R_ are the diffusion coefficient of redox molecules (here *D*_O_ ≈ *D*_R_ = 7.3 × 10^−6^ cm^2^ s^−1^), β is the transfer coefficient (here β ≈ 0.5), *F* is the Faraday’s constant, *R* is the ideal gas constant, *T* is the absolute temperature and *n* is the number of electrons exchanged per molecule (*n* = 1).

**Figure 3:**
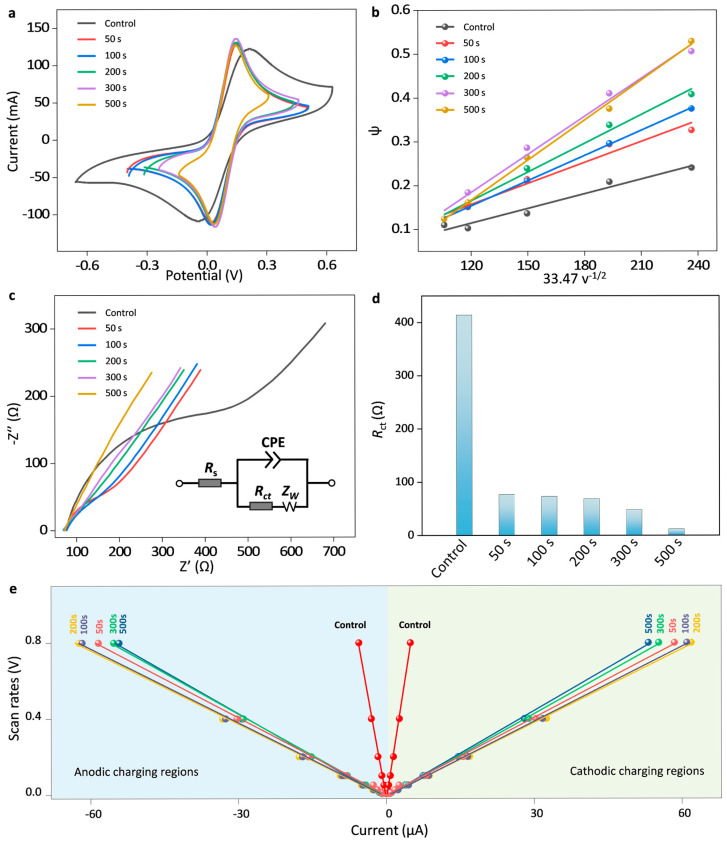
Basic electrochemical performance of SPCEs. (**a**) Cyclic voltammograms of the raw SPCE (control) and AuNPs modified SPCEs at different times of 50 s, 100 s, 200 s, 300 s, and 500 s. (**b**) The corresponding dimensionless kinetic parameter (*ψ*) as a function of the inverse square root of the scan rate (*v*^−1/2^). (**c**) Electrochemical impedance spectra of six SPCEs with equivalent circuit diagram shown in the inset and (**d**) their corresponding interfacial electron transfer resistance (*R*_ct_). (**e**) Anodic and cathodic charging currents of six SPCEs obtained at open circuit potential as a function of scan rate. The average value of the linear slope yields the double-layer capacitance.

Figure 3a shows cyclic voltammograms of the raw SPCE (control) and AuNPs modified SPCEs at different times of 50 s, 100 s, 200 s, 300 s and 500 s, and the corresponding cyclic voltammograms at various scan rates of 20, 30, 50, 80 and 100 mV s^−1^ are provided in Figure S2. The dimensionless kinetic parameter (*ψ*) versus the inverse square root of the scan rates (*v*^−1/2^) is illustrated in Figure 3b and Figure S2. The slope of the linear calibration curve of *ψ* versus 33.47 *v*^−1/2^ yields the values of *k*^0^, during which 33.47 refers to the value of [*πDnF*/*RT*]^−1/2^ (calculated in SI units). The *k*^0^ of AuNPs modified SPCEs at times of 50 s, 100 s, 200 s, 300 s and 500 s was found to be 1.59 × 10^−3^ cm s^−1^, 1.91 × 10^−3^ cm s^−1^, 2.18 × 10^−3^ cm s^−1^, 2.89 × 10^−3^ cm s^−1^, and 3.04 × 10^−3^ cm s^−1^, which is apparently higher than that of the control SPCE (1.16 × 10^−3^ cm s^−1^). It is noted that as the AuNPs deposition time increased, the *k*^0^ showed an upward trend. This is because the prolonged deposition time led to an increase in the number of AuNPs deposited on the electrode surface, thereby enhancing the electrode’s electron conductivity. The improved electron conductivity is beneficial for the interfacial electron transfer, as shown in Figure 3c,d. The equivalent circuit diagram for the AuNPs/SPCE system was shown in the inset, including solution resistance (*R*_s_), charge transfer resistance (*R*_ct_), Warburg resistance (*Z*_w_), and constant phase element (CPE). It was noted that the *R*_ct_ of the AuNPs modified SPCEs at times of 50 s, 100 s, 200 s, 300 s, and 500 s was 78.3, 73.4, 65.8, 69.2, 46.8, and 11.2 Ω, which is much smaller than that of the raw SPCE (422.3 Ω). In addition, the electrochemically active surface area (ECSA) of six SPCEs was directly proportional to the respective double-layer capacitance (*C*_DL_), which was measured at a non-Faradaic potential range with a potential window of typically 0.1 V centered at open circuit potential (OCP). The slope of the linear calibration curve of anodic and cathodic charging currents as a function of the scan rate corresponds to *C*_DL_. The larger the slope, the higher the ECSA of the electrode. Figure 3e illustrates anodic and cathodic charging currents of the raw SPCE (control) and AuNPs modified SPCEs at different times of 50 s, 100 s, 200 s, 300 s, and 500 s. The corresponding cyclic voltammograms of six SPCEs in a non-Faradaic region at various scan rates are provided in Figure S3. It is clear that the ECSA of the AuNPs-modified SPCEs is significantly higher than that of the raw SPCE, indicating that the surface modification of AuNPs could effectively enhance the conductivity of the electrode surface and provide more active sites. However, when the deposition time was extended from 300 s to 500 s, either *k*^0^ or ECSA was only slightly increased, indicating that further prolonging the deposition time has a limited effect on improving conductivity and catalytic activity. Thus, considering both performance enhancement and experimental efficiency, 300 s is selected as the optimal deposition time for subsequent experiments, and the size of AuNPs was counted to be 100 ± 2.3 nm, as shown in Figure S4.

**Figure 4:**
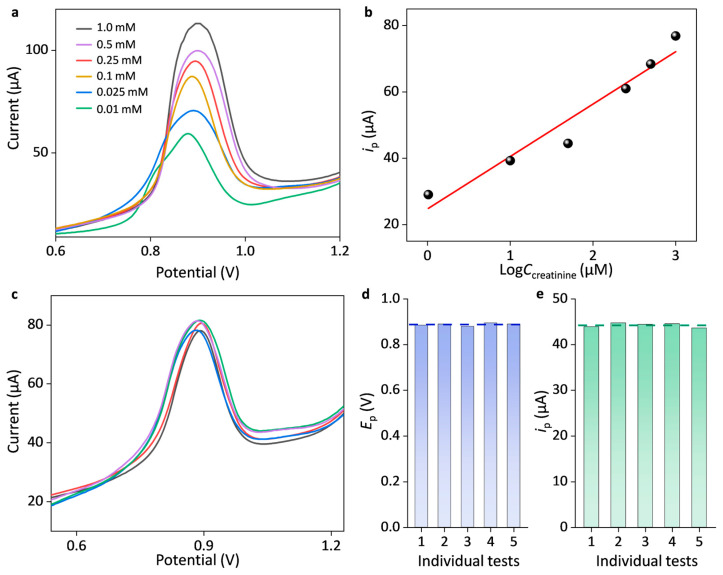
Electrochemical determination of creatinine with the AuNPs-modified SPCE. (**a**) DPV responses in the presence of creatinine with various concentrations of 0.01, 0.025, 0.1, 0.25, 0.5, and 1.0 mM. (**b**) Linear calibration curve of the peak currents versus the logarithm of the concentration. (**c**) DPV responses of five newly prepared AuNPs/SPCEs in the presence of 0.5 mM creatinine, and comparison of their corresponding (**d**) peak potentials and (**e**) peak currents. The electrolyte is 0.1 M phosphate-buffered saline solution.

Figure 4a shows cyclic voltammograms of the optimal AuNPs/SPCE in the presence of creatinine with various concentrations of 0.01, 0.025, 0.1, 0.25, 0.5, and 1.0 mM. The peak current (*i*_p_) of creatinine oxidation was found at the potential of around 0.9 V, and as the concentration increased, the peak current increased accordingly. The *i*_p_ followed a linear calibration relation between 10 μM and 1000 μM with respect to the logarithm of the creatinine concentration, as written by *i_p_ (μA)* = 15.8 lg*C (μM)* + 21.0, *R*^2^ = 0.944 (Figure 4b). The reproducibility of AuNPs/SPCE was performed by five individual newly prepared ones under the same measurement conditions. Figure 4c shows DPV tests of 0.5 mM creatinine measured with five individual AuNPs/SPCEs. These newly prepared sensors exhibited excellent reproducibility, and the standard deviation in the oxidation peak potential and currents is only 0.64% and 0.88%, as, respectively, shown in Figure 4d,e.

**Figure 5:**
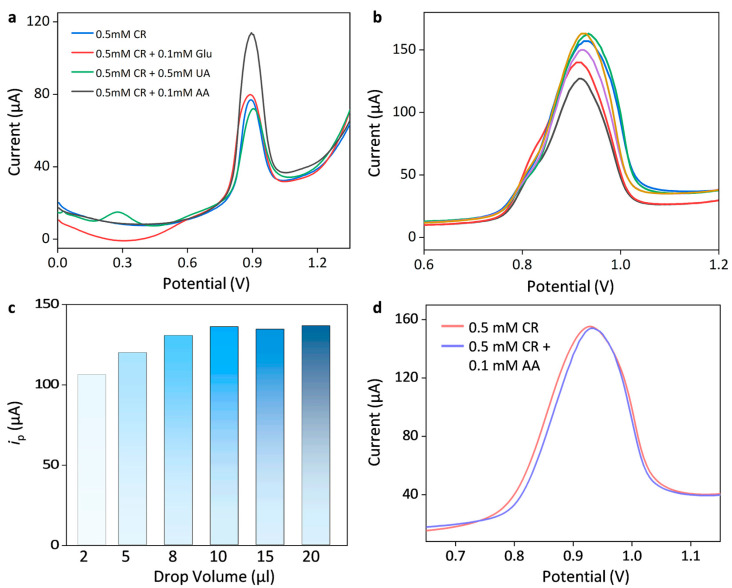
Anti-interference performance for the determination of creatinine (CR). (**a**) DPV responses of 0.5 mM creatinine (CR) in the presence of 0.1 mM glucose (Glu), 0.5 mM uric acid (UA), or 0.5 mM ascorbic acid (AA). (**b**) DPV responses of AuNPs/SPCE toward 0.5 mM creatinine after coating different volumes of Nafion film at 2, 5, 8, 10, 15, and 20 μL (from bottom to top). (**c**) Corresponding peak currents of 0.5 mM creatinine after dropping different volumes of Nafion film. (**d**) Comparison of DPV responses of 0.5 mM creatinine with Nafion/AuNPs/SPCE in the absence and presence of 0.1 mM AA.

Some interference agents, such as glucose, uric acid, and ascorbic acid, possibly produce interfering response currents toward the detection of creatinine. Figure 5a shows DPV responses of 0.5 mM creatinine tested with the AuNPs/SPCE biosensor in the presence of 0.1 mM glucose (Glu), 0.5 mM uric acid (UA), and 0.1 mM ascorbic acid (AA). The choice of concentration of these interference agents results from the upper limit of their physiological concentrations [29,30]. It is noted that the oxidation peak current of UA occurred at the potential range of around 0.3 V, while an apparent oxidation plateau at around 0.6 V corresponded to glucose oxidation. Both UA and Glu had little impact on the determination of creatinine. However, the presence of AA led to a significant increase in the peak currents at around 0.9 V, indicating that the oxidation of AA largely overlapped with the response potential window of creatinine. Thus, it is necessary to eliminate the interfering effects of AA on the quantification of creatinine.

Previous studies demonstrated that proton exchange membranes (such as Nafion membranes) could effectively suppress the interference of substances like AA and enhance electrode selectivity [26]. This is because Nafion membranes consist of a hydrophobic backbone (-CF_2_-CF_2_) and negatively charged hydrophilic sulfonic acid groups (-SO_3_H). Under neutral experimental conditions, AA molecules (pKa = 4.17) exist in the form of anions and would be strongly repelled by Nafion membranes. Thus, the surface modification of Nafion membranes could effectively eliminate the interfering effects of AA. In contrast, creatinine was reported to be positively charged in biological pH conditions, and the motion of charged creatinine molecules would be accelerated toward the negatively charged electrode surface, thereby increasing the flux of creatinine molecules. Figure 5b shows DPV responses of AuNPs/SPCE toward 0.5 mM creatinine after coating different volumes of Nafion membrane, and the peak currents are shown in Figure 5c. The response current of the creatinine first increased with the loading volume of Nafion and then slightly decreased, peaking at 10 μL of loading volume. The initial current rise was attributed to the attractive force of the Nafion membrane that could accelerate the flux of positively charged creatinine molecules toward the electrode surface, leading to the increased current response. As the loading volume further increased, the response current almost leveled off, which was attributed to the counteracting effect between the increased attraction force and the reduced electron conductivity due to the increased thickness of non-conductive membranes. Therefore, 10 μL of Nafion membrane was chosen as the optimal loading volume. Figure 5d shows DPV responses of 0.5 mM creatinine with Nafion/AuNPs/SPCE in the absence and presence of 0.1 mM AA. It is clear that the modification of the Nafion membrane could effectively eliminate interference from AA, while preserving the detection sensitivity of creatinine.

### 3.3. Construction of Multienzyme Cascade Mmodified Biosensor and Its Sensing Performance

Alternatively, we also constructed a typical multienzyme cascade modified creatine biosensor based on the AuNPs/SCPE. The enzymatic creatinine biosensor was gradually established by the electrodeposition of Prussian blue membrane on the optimal AuNPs/SCPE surface, followed by multienzyme cascade modification (which consisted of creatinine amidohydrolase (CA), creatine oxidase (CI) and sarcosine oxidase (SOx)) and drop-casting Nafion membrane to stabilize the sensory interface. Note that during enzymatic creatinine detection systems, the multi-enzyme cascade reaction generates hydrogen peroxide (H_2_O_2_) as a stoichiometric byproduct according to the sequential enzymatic reaction, as described by Equations (4)–(6):

(4) $$\mathrm{Creatinine}+{\text{H}}_{2}\text{O}\overset{\mathrm{CA}}{\to }\mathrm{Creatine}$$

(5) $$\mathrm{Creatine}+{\text{H}}_{2}\text{O}\overset{\mathrm{CI}}{\to }\mathrm{Sarcosine}+\mathrm{Urea}$$

(6) $$\mathrm{Sarcosine}+{\text{H}}_{2}\text{O}+{\text{O}}_{2}\overset{\mathrm{SOx}}{\to }\mathrm{Glycine}+{\text{H}}_{2}{\text{O}}_{2}$$

However, the direct measurement of H_2_O_2_ is compromised by its thermodynamic instability, leading to spontaneous decomposition [31]. To address this limitation, the Prussian blue interlayer was introduced to act as the redox layer. The enzymatically produced H_2_O_2_ oxidized PB to its ferricyanide form, which could then be electrochemically reduced back at a controlled cathodic potential. An apparent redox signal of PB in the presence of 0.1 M PBS solution (pH 7.4) can be observed in Figure S5, during which PB was electrodeposited onto the SPCE surface. This redox cycling strategy enables stable and indirect quantification of H_2_O_2_, thereby providing a reliable measure of creatinine concentration. Compared to direct H_2_O_2_ detection, this approach offered enhanced signal stability by leveraging the rapid electron transfer kinetics and structural robustness of the PB redox layer.

**Figure 6:**
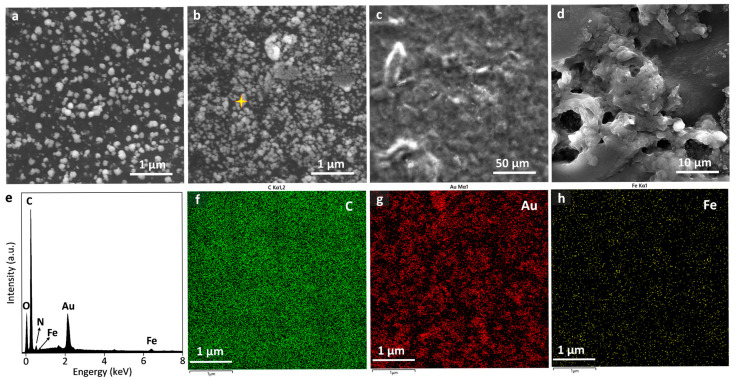
SEM images of (**a**) AuNPs/SPCE, (**b**) PB/AuNPs/SPCE, (**c**) Multienzyme/PB/AuNPs/SPCE, and (**d**) Nafion/Multienzyme/PB/AuNPs/SPCE. (**e**) EDS sampled at the PB/AuNPs/SPCE marked with a yellow star, and (**f**–**h**) elemental mapping of this sensor.

**Figure 7:**
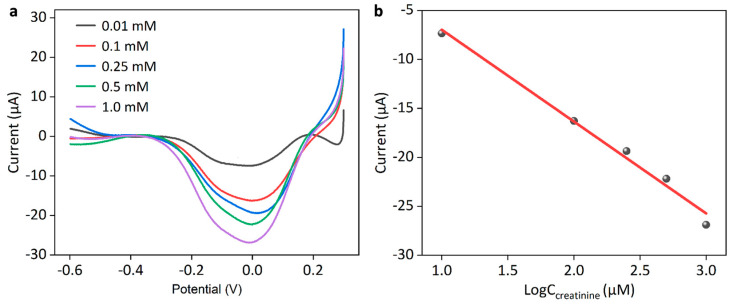
(**a**) Background-subtracted linear sweep voltammograms of creatinine with the Nafion/Multienzyme/AuNPs/SPCE biosensor at different concentrations of 0.01, 0.1, 0.25, 0.5, and 1.0 mM and (**b**) the corresponding linear calibration curve of current responses with respect to the logarithm of the concentration. The electrolyte is 0.1 M PBS.

Figure 6a–d shows the construction of such a composite creatinine biosensor, as shown in SEM images of AuNPs/SPCE, PB/AuNPs/SPCE, Multienzyme/PB/AuNPs/SPCE, and Nafion/Multienzyme/PB/AuNPs/SPCE, respectively. Figure 6e shows the EDS of the PB/AuNPs/SPCE sensor (marked with a yellow star in Figure 6b), and the corresponding elemental mapping is shown in Figure 6f–h, indicating the successful deposition of PB onto the AuNPs/SPCE. Then, the electrochemical quantification of H_2_O_2_ would deduce the concentration of creatinine. In addition, 10 μL of Nafion membrane was loaded to stabilize the composite surface and enhance the anti-interference performance. Figure 7a shows background-subtracted linear sweep voltammograms of creatinine with the Nafion/Multienzyme/AuNPs/SPCE biosensor at different concentrations of 0.01, 0.1, 0.25, 0.5, and 1.0 mM. An apparent reduction signal appeared at the potential range of −0.2 V and 0.2 V, peaking at 0 V. This potential response window corresponded to the reduction of H_2_O_2_ generated by the enzyme cascade-mediated creatinine catalytic reaction. The response current increased with rising creatinine concentration, and the corresponding linear calibration curve of peak currents with respect to the logarithm of the concentration between 10 μM and 1000 μM was shown in Figure 7b, as written by i_p_ (μA) = −9.38 lgC (μM) + 2.4, R^2^ = 0.984. A similar relation of response current to the logarithm of the concentration was also observed with the non-enzymatic AuNPs/SPCE sensor. Specifically, the non-enzymatic sensor detected current signals through the direct oxidation of creatinine, whereas the enzymatic biosensor achieved the indirect detection of creatinine via hydrogen peroxide (H_2_O_2_) generated from a multi-enzyme cascade reaction. Although the non-enzymatic sensor exhibited a relatively higher current response, the enzymatic sensor probably demonstrated superior specificity toward creatinine in biological environments.

**Figure 8:**
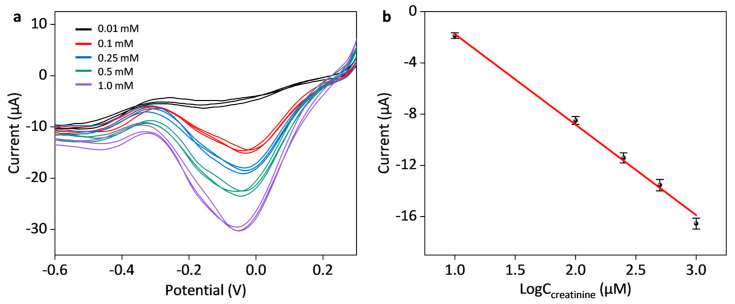
Electrochemical determination of creatinine with different concentrations in human serum. (**a**) Linear sweep voltammograms of creatinine with the Nafion/Multienzyme/AuNPs/SPCE biosensor at different concentrations of 0.01, 0.1, 0.25, 0.5, and 1.0 mM. Each concentration of creatinine was repeated three times, and corresponding voltammograms were overlaid together. (**b**) Linear calibration curve of current responses with respect to the logarithm of the creatinine concentration in human serum.

In order to validate the efficiency and reproducibility of the proposed composite biosensor in real samples, we further carried out repetitive electrochemical measurements of creatinine at different concentrations of 0.01, 0.1, 0.25, 0.5 and 1.0 mM in human serum, during which each concentration was repeated three times and the resulting voltammograms were overlaid together, as shown in Figure 8. During clinical samples, the Nafion/Multienzyme/AuNPs/SPCE biosensor also exhibited increased response currents with increasing concentrations of creatinine, and such response current referred to the reduction of H_2_O_2_ generated by the enzyme cascade-mediated catalytic reaction. Such a composite biosensor showed excellent reproducibility properties for the sensing of creatinine in human serum. The corresponding linear calibration curve of peak currents as a function of creatinine concentration was found to be, i_p_ (μA) = −7.06 lgC (μM) −5.30, R^2^ = 0.996. It is noted that the peak current response of creatinine tested in human serum was lower than that in PBS. The reduction in the current responses might be attributed to two major factors inherent to the complex biological matrix of serum: (i) human serum contains high concentrations of electrolytes (e.g., Na^+^, K^+^, and Cl^−^) and charged biomolecules, which would hinder the interactions between creatinine and the biosensor interface; (ii) the possible non-specific absorption of serum proteins might reduce the effective electrode area and produce competitive steric hindrance effects.

## 4. Discussions and Conclusions

This work reports the fabrication of portable creatinine sensors using commercial screen-printed carbon electrodes (SPCEs) through both non-enzymatic and enzymatic approaches. Electrochemical activation in concentrated acid significantly enhanced the electrochemical performance of the SPCEs, which was further improved following surface modification with Au nanoparticles (AuNPs). The optimal AuNPs-modified SPCE exhibited a 2.6-fold higher electron transfer rate constant (*k*^0^) compared to untreated electrodes. The size of gold nanoparticles was 100 ± 2.3 nm for the optimal AuNPs/SPCE with an electrodeposition time of 300 s. Leveraging the optimal AuNP/SPCE, portable non-enzymatic and enzymatic creatinine sensors were then established. The former one was created by drop-casting a Nafion membrane (Nafion/AuNPs/SPCE), which demonstrated highly selective and sensitive creatinine detection while effectively minimizing interference from physiological levels of ascorbic acid, uric acid, and glucose. An enzymatic biosensor was constructed through sequential modification with Prussian blue (PB) nanoclusters, a multienzyme cascade, and Nafion membrane (Nafion/PB/AuNPs/SPCE), enabling specific creatinine recognition. Both sensors successfully detected creatinine at concentrations ≤ 10 μM, covering the clinically relevant range for both serum and urine analysis. The Nafion membrane significantly enhanced detection selectivity, while the multi-enzyme cascade improved specificity. The AuNPs/SPCE platform showed excellent reproducibility, with standard deviations of only 0.64% for oxidation peak potential and 0.88% for current measurements. Both non-enzymatic and enzymatic creatinine sensors show a positive linear calibration relation of peak currents versus the logarithm of the concentration between 10 μM and 1000 μM, namely, *i_p_ (μA)* = 15.8 lg*C (μM)* + 21.0 and *i*_p_ *(μA)* = −9.38 lg*C (μM)* + 2.4, respectively. The efficiency and reproducibility of the proposed composite biosensor were also confirmed by repetitive electrochemical measurements in human serum.

Future work will focus on expanding creatinine detection to complex biological matrices, including blood, saliva, and urine samples, while simultaneously incorporating urea nitrogen and uric acid as complementary biomarkers for a comprehensive assessment of renal function. This multi-analyte approach will enhance diagnostic accuracy by evaluating key metabolic indicators of kidney health in parallel, enabling more robust clinical correlation and improved disease stratification. The corresponding detection results need to be systematically compared with the clinical gold-standard method to validate the analytical validity and diagnostic accuracy of this approach.

## Data Availability

Data will be made available upon reasonable request.

## Supplementary Materials

The following supporting information can be downloaded at: https://www.mdpi.com/article/10.3390/s25134132/s1. Figure S1: Cyclic voltammograms of different SPCEs at various scan rates of 20, 30, 50, 80 and 100 mV s^−1^; Figure S2: The dimensionless kinetic parameter (*ψ*) of different SPCEs as a function of the inverse square root of the scan rates (*v*^−1/2^); Figure S3: Capacitance measurements of different SPCEs at various scan rates of 0.005, 0.01, 0.025, 0.05, 0.2, 0.4, and 0.8 V/s; Figure S4: Histograms showing the size distribution of gold nanoparticles collected at the optimal AuNPs/SPCE (the electrodeposition time is 300 s) overlapped with Lorentzian fitting curves, with total counts of 210; Figure S5: Cyclic voltammogram of the Prussian blue modified SPCE at a scan rate of 50 mV s^−1^ in presence of 0.1 M PBS solution.
