# Supplementary material for: Electrochemical Determination of Creatinine Based on Multienzyme Cascade-Modified Nafion/Gold Nanoparticles/Screen-Printed Carbon Composite Biosensors
Source: Sensors (Basel). 2025 Jul 2;25(13):4132. doi: 10.3390/s25134132 (PMC12251950; doi:10.3390/s25134132)
Supplement: Supplementary file 1 [file sensors-25-04132-s001.zip › sensors-3634990-supplementary.pdf]

Supplementary File

Electrochemical Determination of Creatinine Based on Multienzyme Cascade-Modified Nafion/Gold Nanoparticles/Screen-Printed Carbon Composite Biosensors

Jialin Yang [1], Ruizhi Yu [1], Wanxin Zhang [1], Yijia Wang [2], and Zejun Deng [1,*]

[1] School of Materials Science and Engineering, State Key Laboratory of Powder Metallurgy, Central South University, Changsha 410083 China
[2] Hunan Key Laboratory for Super Microstructure and Ultrafast Process, School of Physics, Central South University, Changsha 410083, China

*Corresponding authors.
E-mail addresses: zejun.deng@csu.edu.cn (Z. Deng)

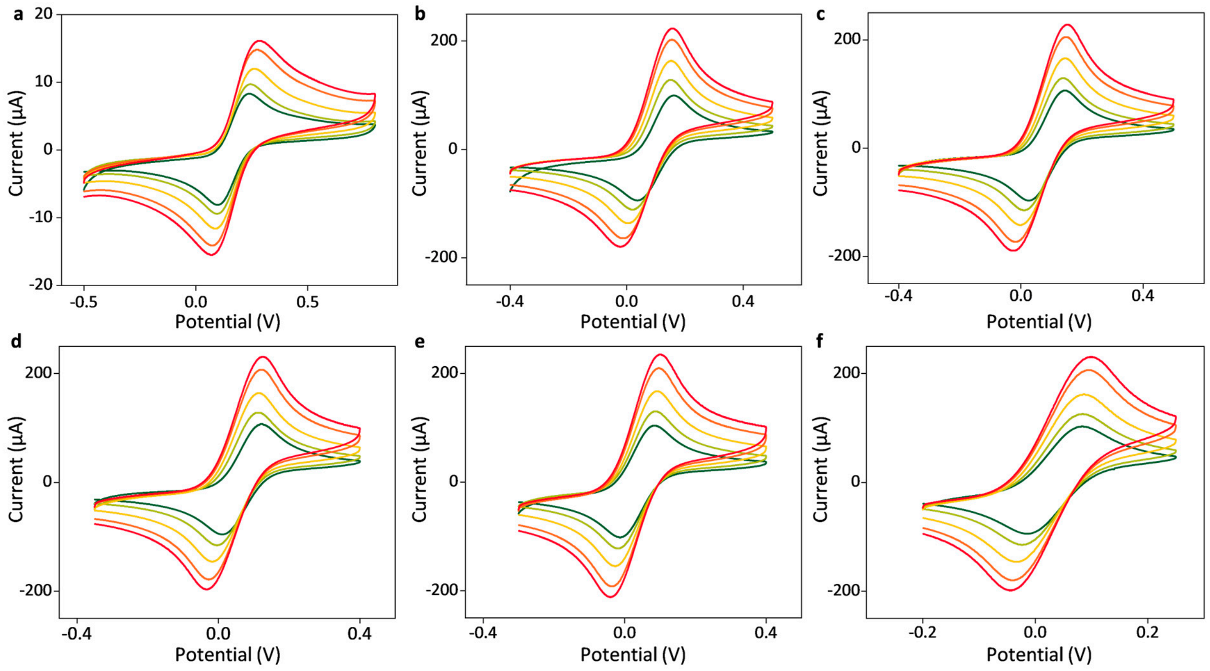

Figure S1. Cyclic voltammograms of (a) the raw SPCE (control) and AuNPs modified SPCEs at different times of (b) 50 s, (c) 100 s, (d) 200 s, (e) 300 s and (f) 500 s at various scan rates of 20, 30, 50, 80 and 100 mV s^{-1}. The solution contains 5 mM $K_3Fe(CN)_6$, 5 mM $K_2Fe(CN)_6$ and 0.1 M KCl.

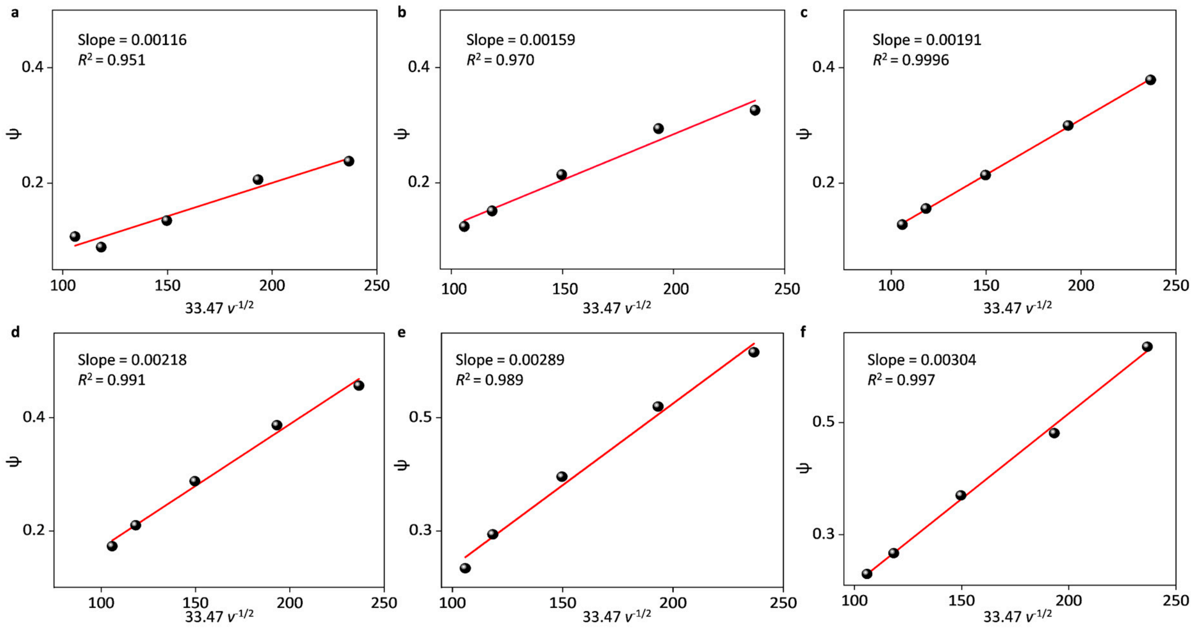

Figure S2. The corresponding dimensionless kinetic parameter (ψ) of (a) the raw SPCE (control) and AuNPs modified SPCEs at different times of (b) 50 s, (c) 100 s, (d) 200 s, (e) 300 s and (f) 500 s as a function of the inverse square root of the scan rates ($v^{-1/2}$).

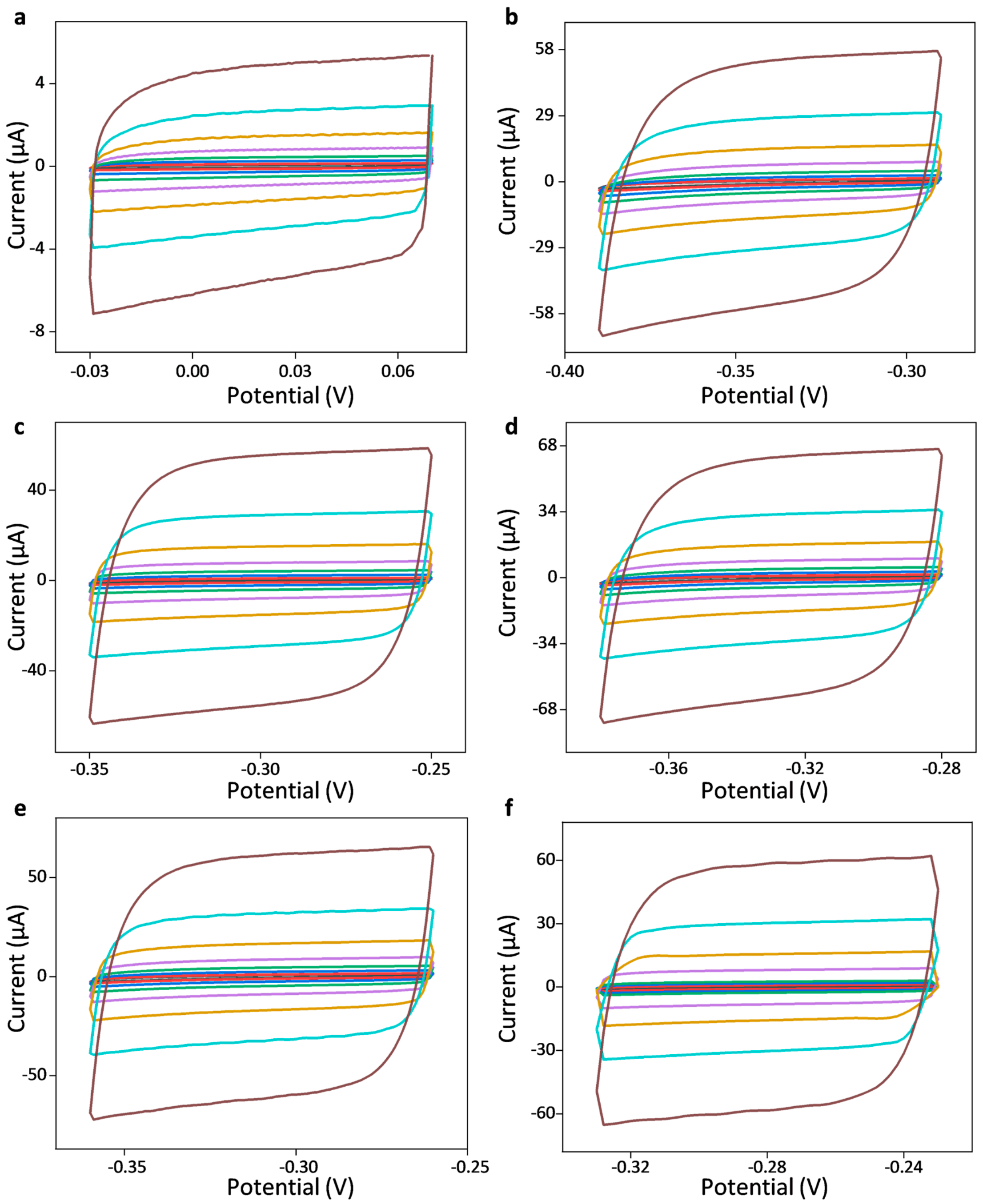

Figure S3. The corresponding cyclic voltammograms of (a) the raw SPCE (control) and AuNPs modified SPCEs at different times of (b) 50 s, (c) 100 s, (d) 200 s, (e) 300 s and (f) 500 s at various scan rates of (from the middle to two ends): 0.005, 0.01, 0.025, 0.05, 0.2, 0.4, 0.8 V/s. The potential ranges swept at at a non-Faradaic potential range with a potential window of typically 0.1 V centered at open circuit potential.

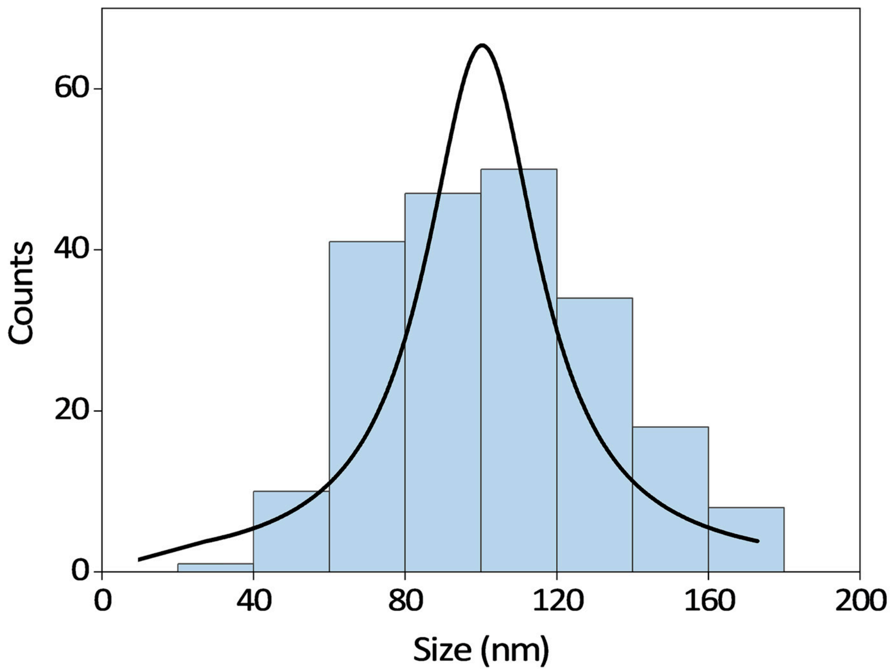

Figure S4. Histograms showing the size distribution of gold nanoparticles collected at the optimal AuNPs/SPCE (the electrodeposition time is 300 s) overlapped with Lorentzian fitting curves, having total counts of 210. The size of gold nanoparticles was obtained to be 100 ± 2.3 nm.

Figure S5 shows CV of Prussian blue modified SPCE at a scan rate of 50 mV s^{-1} in presence of 0.1 M PBS solution. Prussian blue was electro-deposited onto the SPCE via cycling in the range of -0.15 V to 0.3 V in a solution of 2.5 mM $K_3Fe(CN)_6$, 2.5 mM $FeCl_3$, 100 mM KCl and 100 mM HCl. A pair of redox current signal was observed and its reduction signal peaked at around -0.1 V vs. Ag/AgCl.

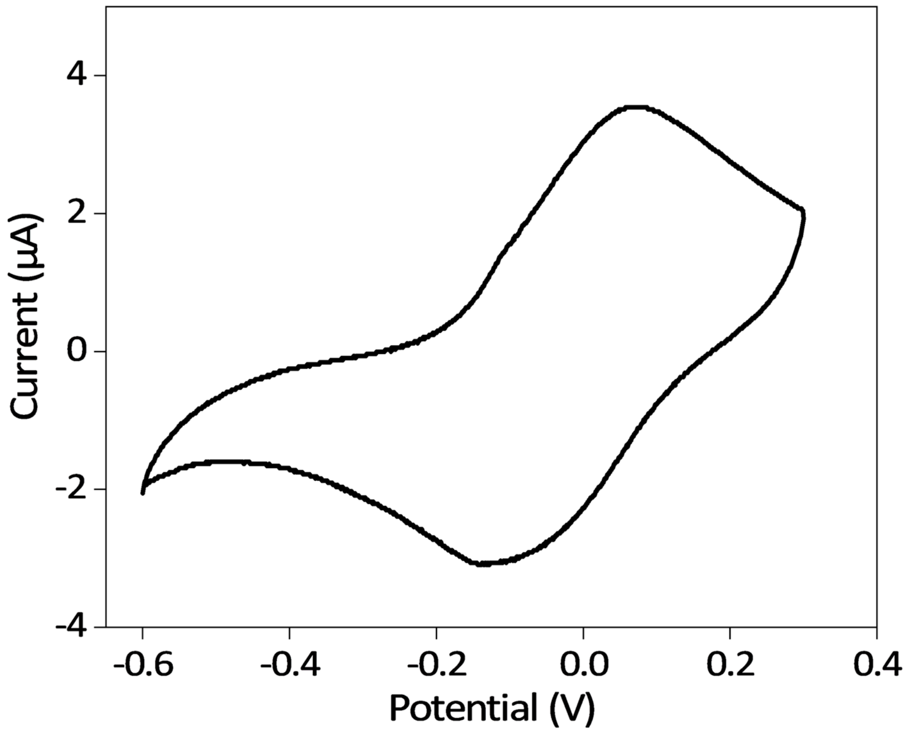

Figure S5. Cyclic voltammogram of Prussian blue modified SPCE at a scan rate of 50 mV s^{-1} in presence of 0.1 M PBS solution.
